# Photobiomodulation with Pulsed and Continuous Wave Near-Infrared Laser (810 nm, Al-Ga-As) Augments Dermal Wound Healing in Immunosuppressed Rats

**DOI:** 10.1371/journal.pone.0166705

**Published:** 2016-11-18

**Authors:** Gaurav K. Keshri, Asheesh Gupta, Anju Yadav, Sanjeev K. Sharma, Shashi Bala Singh

**Affiliations:** Defence Institute of Physiology and Allied Sciences (DIPAS), DRDO, Timarpur, Delhi, India; Massachusetts General Hospital, UNITED STATES

## Abstract

Chronic non-healing cutaneous wounds are often vulnerable in one or more repair phases that prevent normal healing and pose challenges to the use of conventional wound care modalities. In immunosuppressed subject, the sequential stages of healing get hampered, which may be the consequences of dysregulated or stagnant wound inflammation. Photobiomodulation (PBM) or low-level laser (light) therapy (LLLT) emerges as a promising drug-free, non-invasive biophysical approach for promoting wound healing, reduction of inflammation, pain and restoration of functions. The present study was therefore undertaken to evaluate the photobiomodulatory effects of 810 nm diode laser (40 mW/cm^2^; 22.6 J/cm^2^) with pulsed (10 and 100 Hz, 50% duty cycle) and continuous wave on full-thickness excision-type dermal wound healing in hydrocortisone-induced immunosuppressed rats. Results clearly delineated that 810 nm PBM at 10 Hz was more effective over continuous and 100 Hz frequency in accelerating wound healing by attenuating the pro-inflammatory markers (NF-kB, TNF-α), augmenting wound contraction (α-SM actin), enhancing cellular proliferation, ECM deposition, neovascularization (HIF-1α, VEGF), re-epithelialization along with up-regulated protein expression of FGFR-1, Fibronectin, HSP-90 and TGF-β2 as compared to the non-irradiated controls. Additionally, 810 nm laser irradiation significantly increased CCO activity and cellular ATP contents. Overall, the findings from this study might broaden the current biological mechanism that could be responsible for photobiomodulatory effect mediated through pulsed NIR 810 nm laser (10 Hz) for promoting dermal wound healing in immunosuppressed subjects.

## Introduction

Wound healing is a complex, dynamic coordinated cascade of cellular and molecular events that encompasses of four overlapping phases: hemostasis, inflammation, granulation tissue formation and remodeling [1,2]. In pathologic conditions such as non-healing chronic wound, this efficient and orderly process gets hampered which may be the consequences of dysregulated or stagnant inflammation, increased free radicals mediated damage, reduced angiogenesis and decreased collagen accumulation, which prevent the normal processes of wound healing [3,4]. The occurrences of chronic wounds are associated with diabetes, venous stasis, pressure ulcers, burns, frost-bite and military combat injuries [2,5,6].

Chronic wound formation in immunosuppressed subject is a consequence of pathological conditions including autoimmune disorders, immunodeficiency syndrome, hypersensitivity intolerance, asthma and use of drugs such as steroids over a long period in organ transplantation [2,4,7,]. Glucocorticoids (GCs) are a potent class of immunosuppressed compounds that have been used in many pathological conditions [3,7,8]. Pharmacological levels of GCs retard inflammation and wound healing by imparting anti-inflammatory properties, which tend to retard the initial step i.e. chemotaxis of immune cells associated with repair process [4]. The lymphocytopenia, neutrophilia and hampered macrophage activity are signature effects of GC therapy that attenuate or delay the inflammatory phase of wound repair, which consequently causing a decrease in cellular proliferation, neo-vascularization and extracellular matrix (ECM) deposition that ultimately leads to chronic wounds formation [3,4].

Over the years, many approaches for wound care management have been developed, such as use of pharmacotherapeutic agents and bioactive dressings, but their effectiveness are limited [1,4,9]. Therefore, recent extensive research have focused on enhancing the repair process through strategies including the application of tissue engineered scaffolds, gene therapy, stem cell-based therapy and biophysical therapeutic intervention using light-based modality (photobiomodulation, PBM or low-level laser/light therapy, LLLT) that can promote the healing process and save patients from amputation and other severe complications [2,9–12]. So far, the therapies available for wound repair in immunosuppressed subjects are less effective and this has promoted us to consider about PBM therapy for effectual chronic wound care management.

PBM is a potential drug-free, non-invasive application of light widely used for the treatment of a variety of pathophysiological conditions, including healing of chronic wounds, muscles and nerve injuries, reduction of inflammation, pain and restoration of function [13]. In PBM therapy, successful therapeutic outcomes require selection of optimum optical treatment protocols including illumination parameters (such as wavelength, fluence, fluence rate, pulse structure etc.) and treatment regimen. The tissue and light interaction is primarily depends on wavelength, as it determines the absorption mechanism and depth of penetration [14]. It has been reported that cytochrome c oxidase (CCO) is a primary photoacceptor of red and near-infrared (NIR) radiation in mitochondria of cells that activates retrograde light-sensitive cellular signaling events to transport the light signal from mitochondria to nucleus, which eventually alter the cellular metabolism and functions [15]. Both scattering and absorption of light by tissue are highly wavelength-dependent and NIR light around 810–830 nm have been found to have the deepest penetration and homogeneous illumination of the full dermis and part of the hypodermis [12,15].

There are several studies that reported the beneficial effects of NIR 810 nm light for the treatment of different kind of injuries. The PBM of 810 nm irradiation improve neurological performance in traumatic brain injury [16], accelerate both normal and chronic dermal wound healing [17–20] and prevent the appearance of surgical scar [21]. Furthermore, 810 nm laser irradiation exhibited an anti-inflammatory effect on murine bone marrow derived dendritic cells that could be governed by cyclic adenosine monophosphate (cAMP) and reduced nuclear factor (NF)-kB signaling [22]. In another study, it has been clearly shown that 830 nm light can potentially restore the potassium cyanide-inactivated CCO function [23]. The aforementioned findings had encouraged us to design the present study using 810 nm laser, because of its good penetration in tissues and the capacity of this wavelength change the biological signalization of genes and physiological responses, like tissue repair, inflammation and pain control among other responses [12,24,25].

The mode of operation of LLLT can either be continuous or pulsed wave (CW/PW). The recent studies demonstrated that PW does have biological and clinical effects that are different from those of CW. It has been reported that PW is more effective than CW, since there are quench periods (pulse-off times) of longer duration than the on-timings which reduces tissue heating, thereby allowing the use of potentially much higher peak power densities than those used in CW. The biological explanation of the improved effects of PW is either due to some fundamental frequency that exists in biological systems in the range of tens to hundreds of Hz, or alternatively due to some biological process that has a time scale of a few milliseconds [25]. It has been further revealed that LLLT in PW mode can better penetrate through the melanin and other skin barriers, supporting the hypotheses that pulsing is beneficial in reaching deep target tissue and organs [25]. Some recent studies have demonstrated that PW mode is advantageous over the CW mode, particularly, in the context of repair of deep tissue injuries and stroke management [25–27]. Ilic et al., reported that pulsed light produced no neurological or tissue damage, whereas an equal power density delivered by CW caused marked neurological deficits [28]. Although studies has emerged on beneficial effects of 810 nm LLLT for different kinds of injury, however it is not clear whether 810 nm laser irradiation could accelerate dermal wound repair in immunosuppressed subject. Therefore, the present study was undertaken to evaluate the photobiomodulatory effect of 810 nm laser (40 mW/cm^2^; 22.6 J/cm^2^) with CW and PW (10 and 100 Hz) modes on full-thickness excision-type dermal wound healing in hydrocortisone (HC)-induced immunosuppressed rats.

## Materials and Methods

### Animals

Male adult Sprague-Dawley rats (180 ± 20 g; animal colony of DIPAS, Delhi) were used in this study. The animals were maintained under controlled environment at the institute’s animal house at 25 ± 1°C and 12 h: 12 h light: dark cycle. The animals were housed one per cage and fed with granulated food and water *ad libitum*. All animal procedures and experimental protocol were approved by Institutional Animal Ethical Committee (IAEC/DIPAS/2015-03), DIPAS, DRDO (Authorization Number: 27/GO/RBi/SL/99/CPCSEA) and followed the standards set forth in the guide for the care and use of laboratory animals (National Academy of Science, Washington, D.C.).

### Induction of immunosuppressed state

This was achieved by administration of HC (40 mg/kg body wt., intramuscularly) daily for one week prior to wounding, which continued till the end of experiment to maintain the immunosuppressed state of the animals as described earlier [7]. HC was dissolved in sterile pyrogen free water.

### Wound creation

The animals were anesthetized by an intraperitoneal injection of a ketamine-xylazine cocktail (90 mg/kg body wt. ketamine and 10 mg/kg body wt. xylazine) before wound creation and during follow-up treatment. The dorsal surface of the rat was shaved using electric fur clipper, and underlying skin cleaned with 70% ethanol. A full-thickness excision wound (1.5 cm diameter) was created using sterile surgical blade and scissors under aseptic conditions, followed by topical application of pain reliever (Buprenorphine, 0.5 mg/kg body wt.) in order to reduce pain threshold and all efforts were made to minimize suffering. The wound was left uncovered during the whole period of experiment. After 7 days post-wounding, animals were sacrificed using Thiopentone sodium (50 mg/kg body wt., intraperitoneal) and wound tissues were carefully collected to evaluate the biochemical, molecular and histopathological analysis.

### Experimental design

A total of 54 animals were randomly distributed into two groups. The first group comprising of five sub-groups with 6 animals in each: a non-irradiated control immunocompromised group, a CW group, a PW 10 Hz group, a PW 100 Hz group and a silver sulfadiazine group (SSD, 1.0%, w/w, Ranbaxy laboratories Ltd., India) (reference care), was used for the measurement of wound area contraction, pro-healing biochemical markers, histopathological observations and analysis of TNF-α, CCO and ATP levels. The second group comprising of four sub-groups with 6 animals in each: a non-irradiated control immunocompromised group, a CW group, a PW 10 Hz group and a PW 100 Hz group was used to investigate the protein expression levels of matrix metalloproteinases (MMPs), growth factors, cellular and nuclear proteins. HC was administered to all groups daily for 1 week prior to wounding, which continued till the end of experiment to maintain the immunosuppressed state.

### Low-level NIR laser (810 nm) irradiation

**Fig 1:**
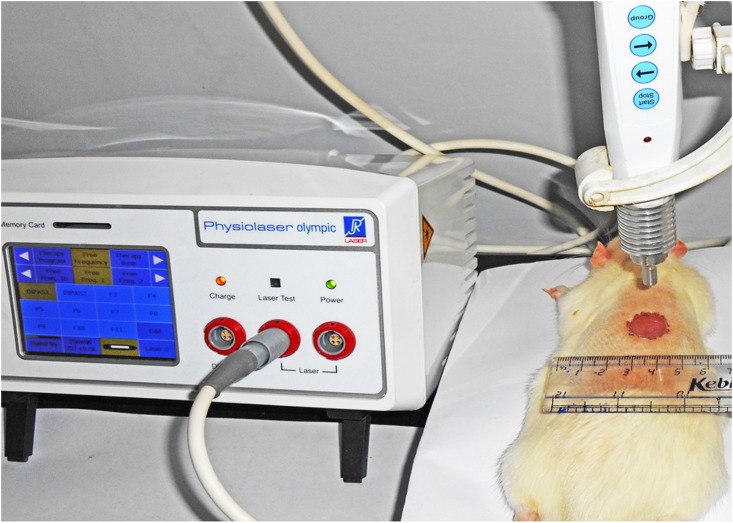
Positioning of the rat and photobiomodulation (PBM) therapy with 810 nm diode laser applied on wound at the dorsal surface of rat without contact. Scale showing the wound diameter size (1.5 cm).

LLLT was performed using an Al-Ga-As diode laser (810 nm, NIR) (Physiolaser Olympic Basic, RJ Laser, Germany) at a laser mode sat on CW, 10 and 100 Hz (duty cycle; 50%) with constant fluence (22.6 J/cm^2^), irradiance (40 mW/cm^2^) and duration (10 min) to deliver a light spot centered on the dorsal surface of the immunosuppressed rat with full-thickness dermal wound. The irradiance was measured using a 3A-ROHS with Nova-II laser power/ energy meter (Ophir Optronics Solutions Ltd., Israel). The aperture and dimension of sensor of power meter was 9.5 mm and 70 mm (width) x 30 mm (depth) respectively. The distance between wound surface and laser probe end point was of 3 cm. The spot of the laser beam had an area of 1.77 cm^2^ on the dorsal surface of the rat (Fig 1). The complete laser parameters are given in Tables 1–3. LLLT was applied once daily on wounds at the same time for 7 consecutive post-wounding days with the first application being 1 h after creation of wound. Non-irradiated control and SSD treated rats were kept anesthetized for the same time period as the laser illuminated rats.

**Table 1:** List of laser parameters of continuous wave.

Laser Parameters	Value
Wavelength	810 nm
Mode	Continuous
Spot size	Diameter = 1.5 cm, area = 1.77 cm^2^
Average irradiance	40 mW/cm^2^
Total fluence	22.6 J/cm^2^
Average power	70 mW
Total energy	28 J
Illumination Time	10 min

**Table 2:** List of laser parameters of Pulse frequency 10 Hz.

Laser Parameters	Value
Wavelength	810 nm
Mode	Pulse frequency 10 Hz
Spot size	Diameter = 1.5 cm, area = 1.77 cm^2^
Average irradiance	40 mW/cm^2^
Total fluence	22.6 J/cm^2^
Average power	70 mW
Total energy	28 J
Illumination Time	10 min
Pulse duration	50 msec
Peak Irradiance	80 mW/cm^2^
Duty cycle	50%

**Table 3:** List of laser parameters of Pulse frequency 100 Hz.

Laser Parameters	Value
Wavelength	810 nm
Mode	Pulse frequency 100 Hz
Spot size	Diameter = 1.5 cm, area = 1.77 cm^2^
Average irradiance	40 mW/cm^2^
Total fluence	22.6 J/cm^2^
Average power	70 mW
Total energy	28 J
Illumination Time	10 min
Pulse duration	5 msec
Peak Irradiance	80 mW/cm^2^
Duty cycle	50%

### Pro-healing parameters

#### Wound contraction and biochemical markers

The wound size was monitored in digital photographs following picture recording. The first photo taken on the day of wounding (day 0) and subsequent photos were captured on the fourth and eighth day post-wounding. A ruler (in millimeter) was placed next to each wound and the scale of the ruler image was set before calculating changes in wound areas in each photograph. Fiji (Image J) software (NIH, Bethesda, MD) was used to assess changes in wound area/initial wound area over time. Values were expressed in square millimeters. The wound tissue excised on the eighth day post-wounding and used to analyze the pro-healing biochemical parameters viz. DNA, total protein, hydroxyproline (HP) and hexosamine (HA) contents [29–32].

### Histological analysis

The wound tissue samples excised on eighth day post-wounding were fixed in 10% buffered formaldehyde, routinely processed and cut into 4-μm sections for hematoxylin and eosin staining and observed for the histopathological changes under a light microscope, followed by photomicrography.

### TNF-α level analysis

The wound tissue homogenate was analyzed using ELISA Kit as per manufacturer’s instructions (Invitrogen Corporation, Camarillo, CA) to determine TNF-α protein content, as described earlier [5]. The color intensity was measured at wavelength of 450 nm. The sample concentrations were calculated from standard curve prepared from recombinant TNF-α. The limit of detection was 11.7–750 pg/mL for TNF-α.

### CCO activity and ATP level analysis

CCO (mitochondrial complex-IV) activity and ATP content in the wound tissue homogenate supernatant (prepared in RIPA buffer) were analyzed using a CCO assay kit (Cytocox1; Sigma-Aldrich, St. Louis, MO, USA) and ATP bioluminescent assay kit (FL-AA, Sigma-Aldrich) respectively as per the manufacturer’s instructions. Briefly, CCO activity was measured by monitoring the decrease in absorbance at 550 nm resulting from the oxidation of ferrocytochrome c to ferricytochrome c by CCO which is located on the inner mitochondrial membrane.

ATP content was quantified by bioluminescent assay measuring light output from luciferin-luciferase reaction. Briefly, 0.1 ml of ATP assay mix solution was added to 96-well luminescence white clear-bottom plate, swirled and allowed to stand at room temperature (RT) for about 3 min so that any endogenous ATP would be hydrolyzed, thereby decreasing the background. The assay began by rapidly adding 0.1 ml of standard or sample and light emission was measured immediately by luminometer (FLUOstar Omega, USA). Luciferin-luciferase luminescent was calibrated versus ATP standards and expressed in relative luminescence intensity.

### Gelatin zymography

Zymography analysis was performed to evaluated MMPs expression in wound tissues, as described earlier [5]. Briefly, samples were homogenized with Tris-buffer (saline 0.9%, Tris 0.05 M, Triton X-100-0.25%, CaCl_2_-0.02 M) and centrifuged for 30 min at 4226 x g at 4°C and supernatants were collected. Tissue extract (50 μg) was subjected to 10% SDS–PAGE containing 0.1% SDS and 1 g/L gelatin under non-reducing conditions without prior boiling. After electrophoresis, gel was washed in 2.5% Triton X-100 for 30 min to remove SDS and allow protein to renature, and subsequently immersed in activity buffer (50 mM Tris/HCl, pH 8.0, 5 mM CaCl_2_, 0.2 M NaCl, 0.02% NaN_3_) for 21–24 h at 37°C. The gel was then stained with 2.5% coomassie brilliant blue-R in methanol, acetic acid and water (4:1:5) followed by destaining with methanol, acetic acid and water (4:1:5). Enzyme activity bands were identified according to their molecular weights. Densitometric quantitative analysis of the protein bands in zymography was performed using Fiji (ImageJ) software program (NIH, Bethesda, MD).

### Western blot for protein analysis

Protein concentrations were determined by the Lowry method [30] and protein from the cytosolic and nuclear fractions of wound tissues were separated by sodium dodecyl sulfate-polyacrylamide gel electrophoresis (10%) and transferred onto nitrocellulose membranes (0.2 μm) (Protran, Whatman, Germany), as described earlier [5]. The membranes were blocked overnight at 4°C in tris buffered saline with Tween 20 (TBST) buffer (0.01 M Tris HCL, pH 7.5, 0.15 M NaCl and 0.05% Tween-20) containing 5% skimmed milk protein. Subsequently, the membranes were probed with primary and secondary antibodies incubated at a dilution of 1:1000 for 2.5 h and 1:10000 for 2 h respectively in 3% skimmed milk protein at RT. The following primary antibodies were used: FGFR-1, fibronectin (FN), HSP-90, HIF-1α, β-actin, α-SM actin, TGF-β2 (#F5421, F3648, H1775, H6536, A1978, A2547; T4442; Sigma-Aldrich), NF-kB (#51–3500, Invitrogen, CA) and VEFG (#SC-152; Santa Cruz Biotechnology, Santa Cruz, CA). The secondary anti-mouse/ rabbit/ goat-IgG antibodies conjugated to horseradish peroxidase (#A9044, A0545, A8919) were obtained from Sigma-Aldrich. The membranes were washed with TBST and immunereactive bands were developed by enhanced chemiluminescent substrate (#CPS1120, Sigma-Aldrich) and exposed to X-ray films (Kodak, Rochester, NY, USA). The band densities were quantified using a scanning densitometric analysis and Fiji (ImageJ) software program (NIH, Bethesda, MD).

### Statistical analysis

The data are expressed as mean ± SEM and statistical significance between experimental and control values were analyzed by one-way analysis of variance (ANOVA) with Dunnett’s post-hoc test using GraphPad Prism 6.0 (GraphPad Software Inc. La Jolla, CA, USA). A value of *p < 0*.*05* was considered as statistically significant. Densitometric data for western immunoblots are expressed as a percentage of the control mean density after normalization to loading controls.

## Results

### Wound contraction after 810 nm LLLT

**Fig 2:**
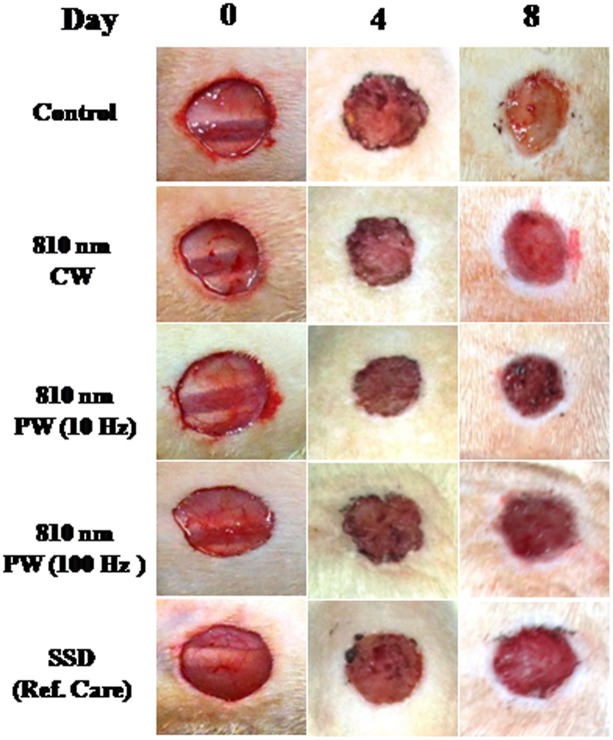
Photomicrographs showing progression during healing of the full thickness dermal wounds in 810 nm LLLT irradiated groups as compared to non-irradiated control and silver sulfadiazine (SSD) ointment (reference care) treated wounds in immunosuppressed rats.

**Fig 3:**
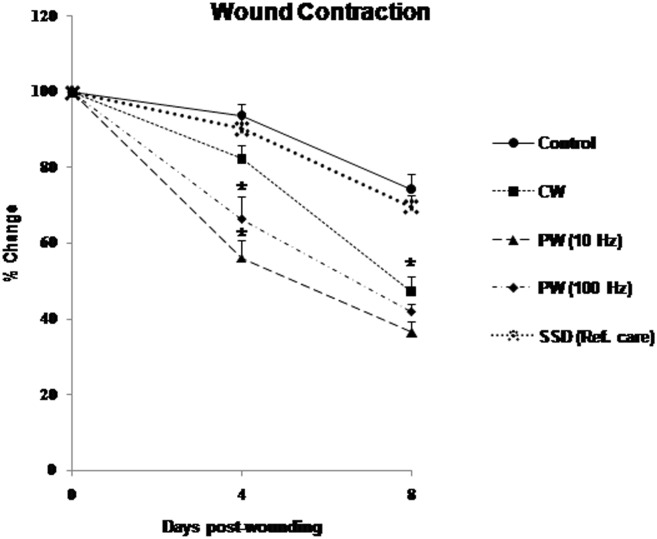
Effect of 810 nm laser on wound contraction rate (% change) in immunosuppressed rats. At days 4 through 8, the 810 nm LLLT (40 mw/ cm^2^, 22.6 J/ cm^2^) 10 Hz group shows a smaller wound area than CW and 100 Hz groups compared to the control. Values are mean ± SEM, n = 6 animals per group. *p < 0.05 compared to the non-irradiated control.

LLLT with 810 nm laser irradiation (40 mW/cm^2^, 22.6 J/cm^2^) with different frequencies (CW, PW 10 and 100 Hz) demonstrated augmented wound healing in HC-induced immunosuppressed rats. Visual inspection of the 810 nm LLLT irradiated wounds showed no evidence of bleeding, exudates, or pus at any time, and all wounds healed without incident. The effects of 810 nm laser irradiation on wound area contraction at fourth and eighth day post-wounding are illustrated in Fig 2. The irradiated animals showed significant faster wound contraction (53–64%, *p* < 0.05) on eighth day post-wounding as compared to the non-irradiated control animals (Fig 3). However, SSD (reference care) treated wound did not show any significant change in wound contraction. This substantiates the healing efficacy of 810 nm LLLT on dermal wounds in immunosuppressed rats.

### Pro-healing biochemical markers after 810 nm LLLT

**Fig 4:**
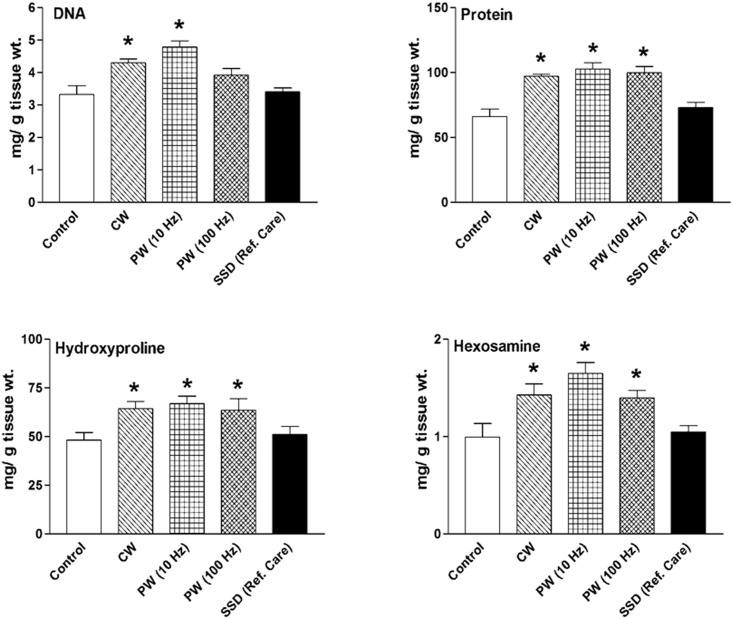
Effect of 810 nm LLLT for 7 days on pro-healing biochemical markers on dermal wound healing in immunosuppressed rats. Values are mean ± SEM, n = 6 animals per group. *p < 0.05 compared to the non-irradiated control.

Fig 4 depicts the effect of 810 nm LLLT with CW, PW 10 and 100 Hz for 7 days post-wounding on pro-healing biochemical markers in dermal wound tissue in immunosuppressed rats. The LLLT increased the cellular proliferation as evidenced by enhanced DNA and total protein contents. The DNA content in CW, PW 10 and 100 Hz groups were significantly *(p < 0*.*05)* increased by 30%, 45% and 19% respectively as compared to the control group. Correspondingly, significant *(p < 0*.*05)* increase in protein content by more than 45% was observed in 810 nm LLLT groups. Similar trend was observed in collagen accumulation and stabilization as evidenced by enhanced HP and HA contents in laser irradiated groups. It showed significant (*p < 0*.*05*) increase by 32–39% and 41–66% in HP and HA contents respectively as compared to the control. Conversely, no significant effects of SSD treatment on pro-healing parameters were noted as compared to the non-irradiated control (Fig 4). Together, these data support the findings that 810 nm laser irradiated dermal wounds exhibited increased mitogenic activity, collagen accumulation and stabilization in immunosuppressed rats.

### Histopathological examinations after 810 nm LLLT

**Fig 5:**
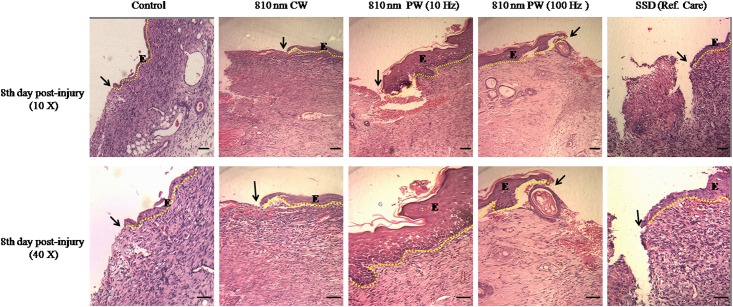
Morphology of the wound skin on eighth day post-wounding in non-irradiated control, 810 nm LLLT irradiated and silver sulfadiazine (SSD) ointment (reference care) treated wounds in immunosuppressed rats. Lower (10X) and higher (40X) magnification showing widespread regeneration of epidermal cells with mature differentiation, greater amount of fibroblast cells, compact collagen deposition, less infiltration of inflammatory cells, new blood vessel formation (angiogenesis) and complete re-epithelialization in 810 nm LLLT irradiated groups, among LLLT irradiated groups these finding was more prominent in PW 10 Hz frequency treated wound. However, non-irradiated control and SSD treatment showed very less epithelial cell regeneration, reduced level of collagen and infiltration of inflammatory cells with less fibroblast. Arrow marked the epithelial migration towards the wound tissue, (E) denotes the skin epithelium and the dotted line illustrate the epithelial margin among groups. Scale bar, 10 μm.

The histopathological assessments of wound tissues removed on eighth day post-wounding of the 810 nm laser irradiated groups, non-irradiated control and SSD treated groups are shown in Fig 5. The semi-quantitative histopathological observations exhibited 810 nm LLLT enhanced fibroblast proliferation, angiogenesis, reduced inflammatory infiltration and re-epithelialization (S1 Table). Histopathological examinations showed more progressive re-epithelialization, thick bundles of well-aligned collagen, reduced congestion, edema and negligible inflammatory infiltration in irradiated groups than non-irradiated control and SSD treated groups. LLLT with 810 nm laser enhanced the formation of granulation tissue in CW, PW 10 and 100 Hz groups, however the regeneration was distinctly enhanced by PW 10 Hz frequency, which showed enhanced fibrogenesis, angiogenesis, collagen deposition and prominent epidermal migration. However, control and SSD treated wounds exhibited increase infiltration of inflammatory cells, few newly formed blood capillaries and fibroblast cells along with loosely packed and reduce levels of collagen fibers with irregular pattern. The histopathological examinations suggest that LLLT with 810 nm laser irradiation promote dermal wound healing by affecting various phases of tissue repair in immunosuppressed rats.

### TNF-α level after 810 nm LLLT

**Fig 6:**
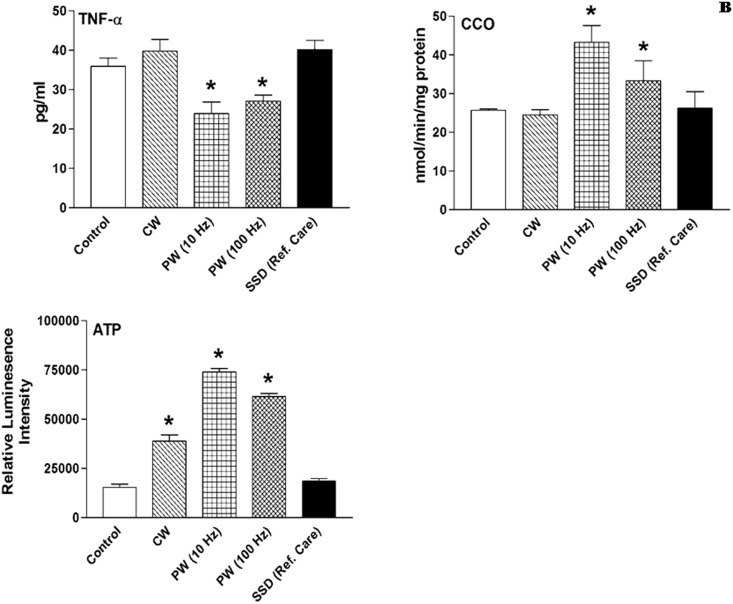
Cytochrome c oxidase (CCO, mitochondrial complex IV) activity, TNF-α and ATP levels in non-irradiated control, 810 nm LLLT irradiated and silver sulfadiazine (SSD) ointment (reference care) treated wounds after 7 days post-wounding in immunosuppressed rats. Values are mean ± SEM, n = 6 animals per group. *p < 0.05 compared to the non-irradiated control.

The level of pro-inflammatory cytokine TNF-α in dermal wound tissue was measured after seven days post-wounding using ELISA after irradiation with 810 nm laser. We found that TNF-α level in the PW 10 Hz group was significantly reduced (33%, *p < 0*.*05*) followed by PW 100 Hz (25%, *p < 0*.*05*) as compared to the non-irradiated control. However, TNF-α level in CW and SSD treated groups were found nearly comparable to the control (Fig 6A).

### CCO activity and ATP content after 810 nm LLLT

CCO activity of mitochondrial respiratory chain was analyzed after 7 days post-wounding irradiation with 810 nm laser. A significant enhanced CCO activity by 68% and 30% (*p < 0*.*05*) was observed in PW 10 and 100 Hz groups respectively as compared to the non-irradiated control. However, CW and SSD groups did not show any significant changes in CCO activity as compared to the control (Fig 6B).

ATP contents were measured in wound tissue to investigate the photobiomodulatory effect of 810 nm laser irradiation on cellular energy status in immunosuppressed rats. The ATP content in wound tissue was significantly (*p* < 0.05) increased in laser irradiated groups (CW, PW 10 and 100 HZ) as compared to the non-irradiated control. In the PW 10 Hz group ATP level was maximized with 3.8-fold increase, however in CW and PW 100 Hz irradiated groups ATP content were enhanced by 1.5-fold and 3-fold respectively as compared to the control (Fig 6C). Conversely, no such change was observed in SSD treated group.

### MMPs expression after 810 nm LLLT

**Fig 7:**
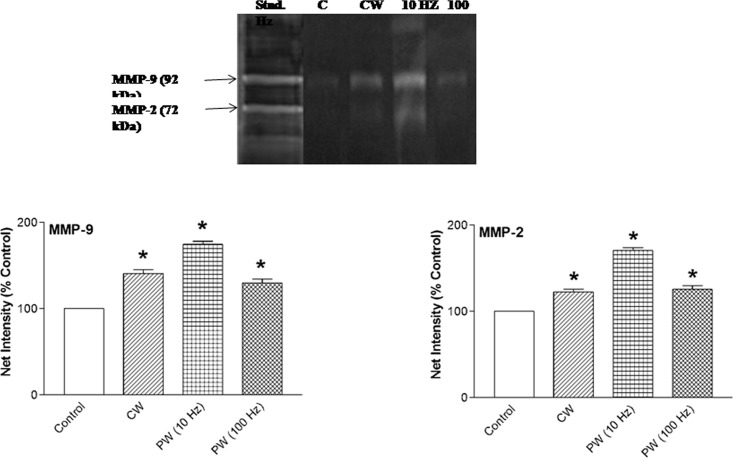
Protein expression of MMP-2 and 9 by gelatin zymography in 810 nm laser irradiated and non-irradiated control wound tissues after 7 days post-wounding. Densitometric analysis using Fiji software. Change in expression expressed as net intensity (% control). Values are mean ± SEM, n = 6 animals per group. *p < 0.05 compared to the non-irradiated control.

The influence of 810 nm LLLT with CW, PW 10 and 100 Hz on extent of tissue remodeling was analyzed by two major MMPs expression viz. MMP-2 (72 kDa) and MMP-9 (92 kDa) after 7 days post-wounding in immunosuppressed rats. Quantitative analysis of band intensity showed that MMP-2 and 9 activities were significantly (*p < 0*.*05*) enhanced in all laser irradiated groups. The most pronounced enhanced expression of MMP-2 and 9 were observed in PW 10 Hz frequency as compared to the non-irradiated control (Fig 7).

### Analysis of growth factors, cellular and nuclear proteins after 810 nm LLLT

**Fig 8:**
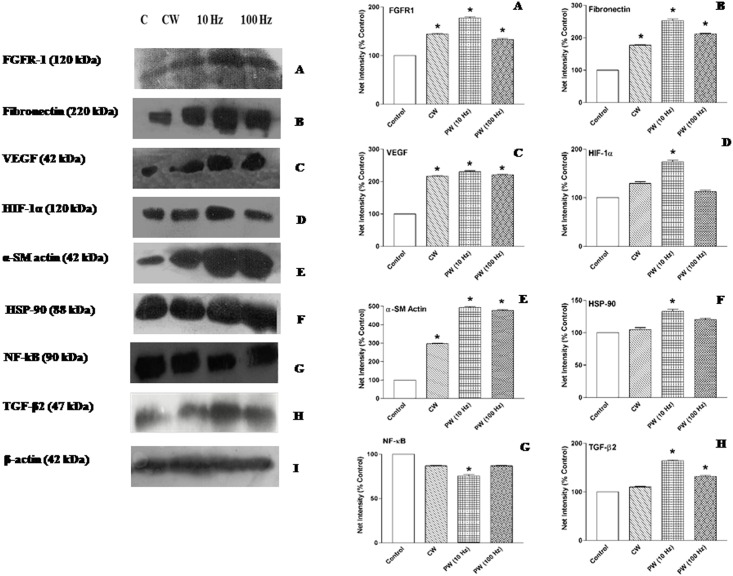
Western blotting assay of the growth factors, extra cellular matrix (ECM), cellular and nuclear protein viz. vascular fibroblast growth factor receptor-1 (FGFR-1), fibronectin, vascular endothelial growth factor (VEGF), smooth muscle α actin (α-SM actin), heat shock protein (HSP)-90, nuclear factor (NF)-kB, hypoxia inducible factor (HIF-1α) and transforming growth factor (TGF-β2) in non-irradiated control, 810 nm LLLT irradiated and silver sulfadiazine (SSD) ointment (reference care) treated wounds in immunosuppressed rats after 7 days post-wounding. Densitometric analysis using Fiji software. Change in expression expressed as net intensity (% control). Values are mean ± SEM, n = 6 animals per group. *p < 0.05 compared to the non-irradiated control.

Fig 8A–8H depicts protein expression profile of growth factors, ECM, cellular and nuclear proteins analyzed by Western immunoblots to elucidate the photobiomodulatory effect of 810 nm laser after 7 days post-wounding in immunosuppressed rats. LLLT resulted in an enhanced expression of FGFR-1 and FN (Fig 8A and 8B) in all laser irradiated groups as compared to the non-irradiated control. The expression of FGFR-1 and FN were maximized in PW 10 Hz group by 77% and 2.5-fold (*p < 0*.*05*) respectively as compared to the control. To assess the extent of neovascularization, the expression of pro-angiogenesis factors, including HIF-1α and VEGF were evaluated after 810 nm laser irradiation. LLLT groups revealed significant increased the VEGF expression (2.1–2.3 fold, *p < 0*.*05*) (Fig 8C), however, HIF-1α expression was significantly up-regulated (70%, *p < 0*.*05*) in PW 10 Hz group as compared to the control (Fig 8D). The α-SM actin correspond the presence of myofibroblasts and its expression is considered to be the characteristic status of wound tissue contraction. The protein expression of α-SM actin significantly enhanced in CW (2.9-fold, *p* < 0.05), PW 10 and 100 Hz (4.9-fold, *p* < 0.05) groups compared to the control (Fig 8E). LLLT had no or minimal effect on HSP-90 expression in CW and PW 100 Hz irradiated groups, whereas PW 10 Hz irradiation conferred a significant up-regulation (30%, *p* < 0.05) as compared to the control (Fig 8F). Remarkably, the expression of NF-kB was found to be significantly down-regulated (25%, *p < 0*.*05*) in PW 10 Hz irradiated group as compared to the control. However, there were minimal or no significant differences observed in CW and PW 100 Hz groups as compared to the control (Fig 8G). The protein expression level of TGF-β2 was significantly (*p* < 0.05) augmented in PW 10 Hz (60%) and 100 Hz (31%) groups as compared to the control. However, CW group did not show any significant change in TGF-β2 expression (Fig 8H).

## Discussion

Acute wound heal through an orderly, highly coordinated series of events, which depends on a dynamic interaction between cells and ECM that promotes swift resolution of inflammation and ingrowth of fibroblasts, keratinocytes and endothelial cells, resulting in functional healing [2]. Conversely, in chronic wound, this orderly progression appears to be halted somewhere in the late inflammatory stage before the initiation of wound closure. This leads to a constellation of cellular and molecular abnormalities, many of which are the result of abnormal cellular-ECM interaction along with increased levels of free radicals and proteases that create an environment of persistent inflammation, resulting in chronic non-healing wounds [2]. The transition of acute wound towards chronic wound is the most common consequences of immune system suppression by the disease itself or prolonged use of corticotherapy. GCs suppressed cell mediated immune response and attenuate the expression of cell adhesion molecules that are essential for the initial phase of healing, which in turn impairs normal cell-mediated immunity at the wound site, causing a significant delay in the healing process [3,4,8].

The biophysical non-invasive PBM approach can be suggested as a positive module for advancement of chronic wound healing. To the best of our knowledge, the present study is first to demonstrate the photobiomodulatory efficacy of NIR 810 nm (Ga-Al-As) laser with CW and PW (10 and 100 Hz) frequency on dermal wound healing in HC-induced immunosuppressed rats. Our current study delineated that PBM with 810 nm laser irradiation (CW, PW 10 and 100 Hz) facilitate enhanced wound healing and attenuated inflammatory response compared to the reference care SSD treated and non-irradiated control groups. The most pronounced stimulation of wound healing was led by PW 10 Hz group, which showed maximal wound area contraction. Our present findings are in conformity with previous reports that NIR laser (808–830 nm) irradiation resulted into faster wound contraction and re-epithelialization in partial and full-thickness dermal wound repair [17,19].

The healing of secondary intention, full-thickness wounds critically depend on granulation tissue formation and wound contraction. Process of wound contraction is characterized by the involvement of a specialized smooth muscle cell, called myofibroblast and their modulation is an important step in wound healing as represented by tissue retraction and fibrosis. The hallmark of the myofibroblastic phenotype is the expression of α-SM actin, which is directly correlated with wound contraction [9]. Our results demonstrated that laser irradiation associated with a striking increase in cellular motility and contractility, this functional transition parallels as increased in expression of α-SM actin.

The dysregulated inflammation assumed to affect subsequent steps of wound repair process that could be detrimental to proper healing and remodeling. NF-kB is a transcription factor regulating expression of multiple genes and has been shown to govern various cellular functions, including inflammation, stress-induced responses and survival. NF-kB activation is regulated by negative feedback mediated by IkB, an inhibitor protein that binds to NF-kB, but can undergo ubiquitination and proteasomal degradation, thus freeing NF-kB to translocate into nucleus and initiate transcription [33]. Activation of NF-kB leads to production of pro-inflammatory cytokine such as TNF-α, produced by activated macrophages, lymphocytes and other cells, which determine the magnitude of inflammation. TNF-α has a critical role in pathogenesis of chronic inflammatory diseases, such as rheumatoid arthritis and local inflammation [34]. The inflammatory response involves various intermediaries and LLLT could manipulate overall NF-kB activation during this process. In the present study, reduction of uncontrolled inflammation in PW 10 Hz group has been linked to down-regulated NF-kB expression. Another factor that may be contributing to the limited inflammation in PW 10 and 100 Hz groups was owing to decreased TNF-α level. As discussed above, LLLT mediated anti-inflammatory effects could be a consequence of NF-kB reduction or inactivation, which in turn reduced the level of TNF-α in laser irradiated wounded tissue, validating the anti-inflammatory notion of 810 nm LLLT. Our results and interpretation are supported by previous evidence in which Assis et al. demonstrated that NIR 808 nm laser at a dose of 180 J/cm^2^, 3.8 mW/cm^2^ decreases NF-kB expression, which led to reduce TNF-α level in cryolesion of tibialis anterior muscle in rats [35]. In another study, LLLT with 80 Hz pulsed NIR 890 nm laser significantly decreased the total number of the mast cells and degranulation during the proliferation and remodeling phase in third-degree burns in rats [10].

The results of present investigation demonstrated that 810 nm LLLT enhanced the cellular proliferation and collagen rich tissue accumulation that provides strength to the regenerated wound in immunosuppressed rats. In the comparative study, among 810 nm laser irradiated groups, PW 10 Hz frequency was most effective as evidenced by augmented cellular proliferation and ECM deposition. However, SSD treated animals did not show any significant changes in pro-healing parameters compared to the non-irradiated control. Consistent with this, histological observations also demonstrated enhanced wound repair as reflected by the reduced infiltration of inflammatory cells, increased neo-vascularization, fibrogenesis, collagen accumulation and faster re-epithelialization in 810 nm laser with PW 10 Hz irradiated group. These findings were in agreement with previous studies which have shown that NIR laser (808–830 nm) irradiation significantly increases granulation tissue formation, fibroblasts and collagen deposition on dermal wounds, which lead to accelerated healing processes [19,20,36].

Wound microenvironment resembles as a hypoxic condition for injured cells that triggers expression of HIF-1α, which generally degraded under normoxic conditions. HIF-1α stabilization under low oxygen resulted in the activation of a transcriptional program, which modulates cellular metabolism, redox homeostasis, vascular remodeling and inflammation [37]. The HIF-1α binds to specific sequences of DNA, which regulate the expression of VEGF thus stimulating angiogenesis. Angiogenesis is a critical component for healing, which is essential for delivery of nutrients and oxygen to injured tissue site. In current study, 810 nm laser irradiation accelerates wound healing by enhancing cellular metabolism and neo-vascularization process. This is likely due to the higher expression of HIF-1α and VEGF by CW, PW 10 and 100 Hz frequency in immunosuppressed rats. It has been previously shown that photobiomodulatory effects of 780 nm laser improves healing of skin flaps by enhancing the amount of new vessels formed in tissue by modulating HIF-1α and VEGF [38].

TGF-β2 is expressed in the early phase of wound healing and is a multi-functional fibrogenic cytokine involved in cellular proliferation, differentiation, ECM deposition, remodeling and other function in wide range of cell types. It has been previously reported that NIR 904 nm superpulsed laser irradiation significantly activates TGF-β2 mRNA expression in MG-63 human osteoblast-like cells [39]. These results suggest that the TGF-β pathway might be a key molecular mechanism triggered by laser irradiation. The result of present study is convincing with these studies showed that 810 nm laser irradiation involved in regulating ECM-remodeling by up-regulation of TGF-β2 expression in PW 10 and 100 Hz groups and thus promote cell proliferation and migration of dermal fibroblast.

HSP-90 expressed constitutively under normal conditions to maintain protein homeostasis and is induced upon environmental stress. HSP-90 has a limited subset of substrates, most of which are signaling molecules [40]. The biological functions of HSP-90 go beyond their chaperone activity. They are essential for maturation, structural maintenance and proper regulation of target proteins and other signaling molecules. The role of HSP-90 has been shown in wound healing under the influence of HIF-1α. Increased expression of HIF-1α in turn causes exocytosis of HSP-90 to ECM, promotes the migration of skin fibroblasts, which further facilitate the healing processes in laser irradiated rats [5]. In this study, we found that PW 10 Hz laser irradiation enhanced the expression of HSP-90 at wound site and thus promotes wound repair processes.

Fibroblast growth factors (FGFs) and their receptors (FGFRs) have been implicated in a variety of important biological processes such as mitogenesis, angiogenesis, cell migration, differentiation, mesoderm induction bone growth and limb development. FGFR-1 is a membrane-spanning tyrosine kinase that serves as a high-affinity receptor for FGFs. FGFR-1 is highly expressed in developing human tissues including vascular basement membranes, brain and skin [6]. The up-regulated expression of FGFR-1 indicate that 810 nm LLLT promote wound healing in immunosuppressed rats by enhancing the proliferation of cells in chronic wound. The PW 10 Hz frequency was more effective than CW and PW 100 Hz as compare to the non-irradiated control.

FN is a multi-functional; ECM glycoprotein can be a ligand for cell surface, collagen, fibrinogen or fibrin, glycosaminoglycans, proteoglycans and heparin. FN mediates a wide variety of cellular interactions with the ECM and plays important role in cell adhesion, migration, growth and differentiation [41]. Irradiation with 810 nm laser significantly accelerates healing, cellular proliferation and migration in immunosuppressed rats as reflected by enhanced FN expression in comparison to the non-irradiated control. This finding was in line with observation of Kim et al., who reported that 808 nm LLLT caused significant increase of FN and type-I collagen levels after corticision around a moving tooth [11].

MMPs play an important role in regulating tissue repair processes such as removal of necrotic tissue, elimination of damage proteins, destruction of provisional ECM, facilitate cell migration and regulate the activity of some cytokines. Various kinds of cells such as fibroblast, smooth muscle, endothelial, epithelial and inflammatory cells are responsible for its synthesis and secretion at wound site. The result of present study indicates that laser irradiation with CW and PW frequency accelerates the recovery of dermal wound injury in immunosuppressed rats. This recovery was accompanied by increased expression and activities of MMP-2 and 9. This finding is in line with previous report that demonstrated the involvement of MMP-2 in 830 nm LLLT regulated muscle regeneration process [42].

The absorption of photons from red and NIR laser occurs at the terminal enzyme CCO in mitochondrial respiratory chain (complex-IV), that leads to electronically excited states and resulting into quickening of electron transfer reactions [15]. More electron transport inevitably causes increased production of ATP [43]. Cells require an input of free energy (ATP) for the maintenance of highly organized structures, synthesis of cellular components, generation of electric impulse and for many other processes. The activation of CCO leads to several downstream events, such as increased cellular metabolic activity, production of ATP and cell proliferation. Increased activity of CCO in 810 nm laser irradiated groups with PW 10 and 100 Hz mode substantiate the activation of CCO by PBM, which in turn enhanced the ATP production and specifies that 810 nm LLLT up-regulates the mitochondrial respiration. Our findings made synchronization with previous studies, which declared increased CCO activity and ATP synthesis in leg, gluteus and lower-back muscles of mice following light-emitting diode therapy (LEDT) using a LED cluster (630 nm and 850 nm, 80 mW/cm2, 7J/cm2) led to increased muscle performance [44]. The 10 Hz frequency showed maximum CCO activity and ATP production over other laser irradiated groups, this may be owing to greater absorption of 810 nm photons by mitochondrial complex-IV at 10 Hz. The earlier finding also corroborate our results and suggest that laser irradiation at 10 Hz mode was most effective for improving neurological outcome in traumatic brain injury and proposed the possible hypothesis that occurrence of positive resonance between the frequency of the pulsed (4–10 Hz) light and brain wave [25]. Increase production of ATP endorsed by in-vitro finding of Huang et al., who evaluated neurons protection ability of 810 nm laser against excitotoxicity in primary cortical neurons [45].

## Conclusion

**Fig 9:**
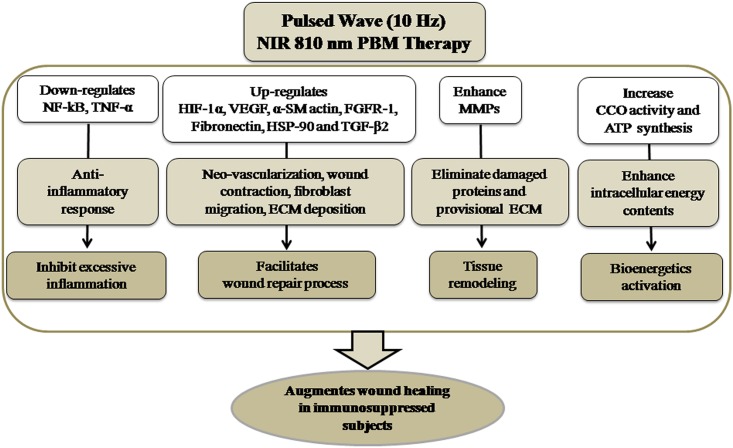
Mechanism of action of photobiomodulatory effects of 10 Hz-pulsed wave 810 nm (NIR) laser showing anti-inflammatory effects, an enhance cellular proliferation, neo-vascularization, ECM accumulation in conjunction with tissue remodeling and bioenergetics activation, which collectively augments dermal wound healing in immunosuppressed rats.

Taken together our findings indicate that PBM with 810 nm laser inhibit excessive inflammation, facilitate cellular proliferation, ECM accumulation, tissue remodeling and bioenergetics activation, which in turn influence the augmented dermal wound healing in immunosuppressed rats (Fig 9). In Addition, healing efficacy of 10 Hz pulsed mode was more promising than CW and 100 Hz pulsed mode. Accordingly, current data may facilitate the understanding of wound healing mechanisms mediated trough NIR pulsed 810 nm LLLT in immunosuppressed rats.

## Supporting Information

S1 Table Semi-quantitative histopathological findings of the skin wound on eight day post-wounding in non-irradiated control, 810 nm LLLT irradiated and silver sulfadiazine (SSD) ointment (reference care) treated wounds in immunosuppressed rats. (DOCX)
