# Supplementary material for: Photobiomodulation with Pulsed and Continuous Wave Near-Infrared Laser (810 nm, Al-Ga-As) Augments Dermal Wound Healing in Immunosuppressed Rats
Source: PLoS One. 2016 Nov 18;11(11):e0166705. doi: 10.1371/journal.pone.0166705 (PMC5115773; doi:10.1371/journal.pone.0166705)
Supplement: S1 Table — (DOCX) [file pone.0166705.s001.docx]

| **Groups** | **Congestion** | **Edema** | **Infiltration** | | **Fibroblast** | **Angiogenesis** | **Epithelialization** |
| --- | --- | --- | --- | --- | --- | --- | --- |
|  |  |  | **PMNLs** | **Monocytes** |  |  |  |
| **Control** | **+++** | **+++** | **++** | **+++** | **+** | **+** | **+** |
| **810 nm CW** | **-** | **-** | **++** | **+** | **++** | **+** | **+** |
| **810 nm PW (10 Hz)** | **-** | **-** | **+** | **+** | **+++** | **+++** | **+++** |
| **810 nm PW (100 Hz)** | **-** | **-** | **+** | **+** | **++** | **++** | **++** |
| **SSD**  **(Ref. Care)** | **++** | **+++** | **++** | **++** | **+** | **++** | **+** |

**S1 Table**

Semi-quantitative histopathological findings of the skin wound on eight day post-wounding in non-irradiated control, 810 nm LLLT irradiated and silver sulfadiazine (SSD) ointment (reference care) treated wounds in immunosuppressed rats.

+, Slight; ++, Moderate; +++, Marked; –, Absent; PMNL, polymorphonuclear leukocyte.
